# Sensory and Physical Characteristics of *M. biceps femoris* from Older Cows Using Ginger Powder (Zingibain) and Sous Vide Cooking

**DOI:** 10.3390/foods10081936

**Published:** 2021-08-20

**Authors:** Zahra B. Naqvi, Peter C. Thomson, Michael A. Campbell, Sajid Latif, Jerrad F. Legako, David M. McGill, Peter C. Wynn, Michael A. Friend, Robyn D. Warner

**Affiliations:** 1 School of Agricultural, Environmental and Veterinary Sciences, Charles Sturt University, Boorooma St., Wagga Wagga, NSW 2678, Australia; mcampbell@csu.edu.au; 2 Graham Centre for Agricultural Innovation, Albert Pugsley Place, Wagga Wagga, NSW 2678, Australia; peter.thomson@sydney.edu.au (P.C.T.); slatif@csu.edu.au (S.L.); pwynn@csu.edu.au (P.C.W.); 3 School of Life and Environmental Sciences, The University of Sydney, Sydney, NSW 2006, Australia; 4 National Life Sciences Research Hub, Charles Sturt University, Wagga Wagga, NSW 2795, Australia; 5 Department of Animal and Food Science, Texas Tech University, Lubbock, TX 79409, USA; Jerrad.Legako@ttu.edu; 6 Faculty of Veterinary and Agricultural Sciences, The University of Melbourne, Parkville, VIC 3010, Australia; david.mcgill@unimelb.edu.au (D.M.M.); robyn.warner@unimelb.edu.au (R.D.W.); 7 Office of the Pro Vice-Chancellor (Research and Innovation), Charles Sturt University, Locked Bag 588, Wagga Wagga, NSW 2678, Australia; mfriend@csu.edu.au

**Keywords:** beef, sensory, physiochemical, older animals, zingibain, toughness

## Abstract

This study aimed to evaluate the sensory and physical characteristics of zingibain-injected meat combined with sous vide cooking. *M. biceps femoris* (BF; *n* = 12) acquired from 6–7 year old Angus cows were cooked using the sous vide method at 65 °C, for 8 h or 12 h, either with ginger powder (GP) injected in a 2 g/L solution in water (treatment) or un-injected (control). The sensory attributes included flavour, juiciness, tenderness, and physicochemical characteristics were Warner-Bratzler shear (WBSF), hardness, total water content (TWC), cooking loss (CL) and collagen content. A significant improvement in tenderness with injection treatment and cooking time was observed, as evaluated through trained sensory panellists, and reduced WBSF and hardness (*p* < 0.05 for all). The flavour of the meat was not affected by injection treatment or cooking time (*p* > 0.05), but juiciness and TWC were reduced with longer cooking times (*p* < 0.01 for both). Soluble collagen increased with injection treatment and cooking time (both *p* < 0.05). Moderate to high correlations were found between sensory and physical measurements for tenderness and juiciness. The longer cooking time (12 h) with GP injection treatment caused over tenderization of the meat. The soft texture associated with over-tenderization may be suitable for some specialised consumer markets, for instance, the elderly population with chewing difficulties. Improving the eating quality of low-quality meat from old animals through sous vide cooking and the use of ginger proteases may increase the acceptability of lower value beef, potentially enhancing the commercial value of carcasses typically produced in the beef industry.

## 1. Introduction

A consumer’s acceptance of and preference for purchased meat largely depends on sensory experience in consuming the cooked product. Sensory attributes include all aspects of tenderness, juiciness, and flavour of meat. Tenderness is widely considered as the most important eating quality attribute associated with consumer preferences [1]. Physical qualities of the meat such as visual appearance, colour, freshness, fat content, and some extrinsic factors including price and brand influence a consumer’s decision to purchase meat at the retail level [2,3]. Sensory attributes of the cooked meat, however, mainly tenderness, play an important role in its acceptability and the decision to repurchase the meat [4,5]. Consumers are willing to pay premium prices for beef if tenderness and high overall eating quality can be guaranteed [6,7]. Provision of consistent texture and good eating experience to consumers has been a challenge to the beef industry often due to the inclusion of older, cull animals in the beef value chain. Meat from older cows is tougher and has inferior eating quality compared to that from younger animals [8]. The toughness in meat from old animals is due to the maturation of crosslinks in collagen which results in the strengthening of intramuscular connective tissue [9,10]. The strength of connective tissue varies between carcasses, and muscles within a carcass, largely due to species, breed of animal, age, nutritional history and cooking conditions [11,12,13]. Improving the consumer experience of tough or lower quality meat requires value-adding, which has the potential to increase the profitability of the beef industry.

The use of exogenous proteases derived from plants (papain, bromelain, actinidin, ficin and zingibain), bacteria (collagenases, bacillus protease), and fungi (aspartic protease) improves the tenderness and eating quality of meat [14,15,16,17,18,19,20,21]. Ginger rhizomes (*Zingiber officinale* Roscoe) contain a protease ‘zingibain’ that belongs to the peptidase family of cysteine proteases [22]. It has significant proteolytic activity and therefore can be effective in tenderizing tough meat [23,24,25]. It improves tenderness [26,27] and sensory attributes of cooked meat [28,29,30]. The use of fresh ginger extract can, however, produce a pungent odour causing off-flavour in treated meat [9,31]. In our previous study in which we investigated the impact of injection of ginger powder (GP) solution containing partially purified zingibain in meat from older animals on eating quality, we found improvement in tenderness and quality of meat [21]. This has led to this further investigation of the effects of this technique on sensory characteristics and flavour of beef. 

Sous vide is a low-temperature long-time (LTLT) cooking method that involves cooking of vacuum packed meat at a precisely controlled temperature and time in a water bath followed by rapid cooling [32]. The food industry is increasingly adopting this method due to several advantages it provides over conventional cooking methods [33]. It offers a uniform texture and improved tenderness particularly for low-value tough meat [34,35,36,37]. Past research has reported improved sensory characteristics, more appealing texture and controlled doneness of the meat compared to several conventional cooking methods [38,39,40]. Sous vide cooking coupled with protease marination, including with ginger proteases, has been studied to improve texture and physical characteristics of the meat [16,17,21,41]. The combined effect of sous vide cooking together with treatment with proteases from injecting ginger powder containing zingibain on the sensory characteristics of meat has not been studied previously [21]. The current study was designed to evaluate the sensory and physical characteristics of meat from older cows using both ginger powder injection and sous-vide cooking.

## 2. Materials and Methods

The experiment was conducted on *biceps femoris* muscles (*n* = 12) obtained from six to seven-year-old Angus cows which were offered a concentrate ration for 56 days before slaughter. Concentrate feeding of the cattle was undertaken at Charles Sturt University, Wagga Wagga, NSW, Australia, in 2019. The concentrate ration was formulated to contain approximately 15% crude protein, approximately 40% neutral detergent fibre and a minimum of 10.5 MJ metabolizable energy (ME)/kg DM using 12.3% wheaten stubble, 11.9% barley hay, 70.8% pre-mix pellets (Oilseed Australia Pty Ltd. Melbourne, Australia), and 5% canola meal (Riverina Oils & Bio Energy Pty Ltd. Wagga Wagga, NSW, Australia). All ingredients were used on a total ration dry matter basis. The cows were kept in lairage with no access to feed and *ad libitum* access to water on the night before slaughter, at the Teys Australia™ abattoir at Wagga Wagga. After chilling of carcasses at 2–3 °C for 24 h, the *Biceps femoris* (BF) muscles were removed from the hindquarter, vacuum-packed stored for 24 h at 2–3 °C and afterwards transported to the meat laboratory at Charles Sturt University, Wagga Wagga. All BF were aged for 14 days at 4 °C and then stored at −20 °C until further processing. 

### 2.1. Injection Treatment and Cooking Process

BF were thawed in a cool room at 2–4 °C for 48 h. After thawing, BF were cut into halves parallel to muscle fibres after removing visible surface fat and connective tissue. Each half was randomly allocated to injection treatment; control no injection (*n* = 12), or injection treatment (*n* = 12). The treatment pieces were injected to 116% raw weight (weight + 16%) with a ginger powder-based solution at a concentration of 2 g/L ginger powder in water (room temperature) while the control samples were vacuum packed and placed in a 4 °C cool room. The ginger powder ‘DigestEasy’ was obtained from Biohawk (QLD, Australia). The presence of zingibain protease in the ginger powder solution was previously analysed using sodium dodecyl sulphate–polyacrylamide gel electrophoresis (SDS PAGE) [21]. Ginger powder solution was injected manually using a needle (1.2 mm × 38 mm: BD PrecisionGlide^TM^, Franklin Lake, NJ, USA). Multiple injections were applied to the muscle with each injection volume of 3–4 mL, equidistant (approximately 2–2.5 cm apart), and 3–4 cm deep into the muscle. After injection, muscles were vacuum-packed and stored at 4°C for 24 h. The next day, both control and injected meat halves were subsequently cut in half perpendicular to the direction of muscle fibres, into two steaks of 2.5 to 3 cm thickness which were randomly selected for sous vide cooking times of 8 h (*n* = 12) or 12 h (*n* = 12). Cooking of samples in a vacuum-packed plastic bag was performed in a circulating water bath (FusionChef™, Julabo Diamond XL, Julabo GmbH, Seelbach, Germany) set at a temperature of 65 °C. The internal temperature of a random sample in a water bath was monitored with a thermo-probe attached to the water bath (FusionChef ™, Julabo, Germany). The probe was inserted through foam sealing tape (Julabo Diamond XL, Julabo GmbH, Seelbach, Germany) on the vacuum bag to retain the vacuum conditions in the bag. After cooking, the vacuum bags were opened, and the cooking loss fluid was saved in a 10 mL plastic tube, then frozen at −20 °C until processed to assess soluble collagen content. After cooking, the steaks were then used either by the trained sensory panel, or for physiochemical measurements, outlined below.

### 2.2. Trained Sensory Evaluation

The Charles Sturt University Human Research Ethics Committee approved the project (Protocol number: H20315) for trained sensory panel evaluations. A group of 12 participants who regularly consume beef meat were recruited on a voluntary basis. The panel included a diverse range of age, gender and ethnic backgrounds. A lexicon to evaluate the sensory characteristics of the meat based on the research objectives was adopted [42,43], shown in Table 1. Participants were trained in a selected lexicon following the research guidelines provided by AMSA [43]. A total of seven training sessions, each of 1.5 h duration, were conducted to achieve a satisfactory level of participant confidence in evaluating the sensory attributes of the meat. During training sessions, a range of reference samples according to the selected lexicon (Table 1), with varying tenderness, juiciness, flavour and texture, were provided to participants. Meat for preparation of reference samples was purchased from a local butcher, as described in Table 1. Following the 7 training sessions, three experimental panel sessions were conducted to evaluate the prepared meat samples. Two panel sessions were conducted on one day, a morning and an evening session, and the third session was conducted on the following day. 

Each freshly cooked sample steak was cut into six samples, each of around 2.5 cm^3^ and placed into a 60-mL cup with a lid (purchased from local market). Each sample was given a unique code labelled on the lid of the cup. A plan for randomization of all samples to all sessions was generated using the statistical package R with a user-written script. A total of eight samples were presented to each participant in each session. Samples were served between 60–65 °C to participants immediately after cooking. During testing, each of the panellists were seated on a separate table in a sensory testing room. Panellists were provided with a bottle of water, unsalted crackers (palate cleanser), toothpicks, napkins, wooden fork, paper-based form and a ballpoint pen. For each sample, tenderness, juiciness, flavour, and textural attributes were evaluated on a line scale having eight points, with descriptions for each scale number as shown in Table 2. Panellists were asked to count the number of chews needed to ingest each sample and record it. They were also asked to describe the meat texture of each sample and were given three options of ‘mealy’, ‘mushy’, or ‘other’, and were required to select one of the three options.

### 2.3. Physicochemical Measurements

The physicochemical measurements including cooking loss, total water content, Warner-Bratzler shear force (WBSF), texture profile analysis (TPA) and total and soluble collagen were conducted on the cooked meat samples, whereas the total water content (TWC) of both raw and cooked meat samples were determined. These physiochemical measurements were conducted on all treatments within each muscle, across the muscles, resulting in a replication of *n* = 6 for all measurements for each treatment.

#### 2.3.1. Total Water Content and Cooking Loss

TWC was measured, following the method of Oillic et al. [44], on 24 samples, comprising six replicates of each treatment, injection treatment (control and GP injection) and cooking time (8 h, 12 h). Briefly, a meat sample (3–5 g) was placed in a hot air oven (N759, S.E.M, Australia) at 104°C for 48 h. After cooling to 25 °C the samples were placed in a desiccator, and their weight was measured to calculate the percentage of weight loss relative to the weight of samples before oven drying. For cooking loss, the meat samples after 24 h injection treatment were opened and dried with a paper towel and the initial weight was measured before cooking. Similarly, samples were weighed after cooking. Cooking loss was calculated as a weight difference before and after cooking relative to the weight of the meat before cooking and expressed as a percentage.

#### 2.3.2. WBSF and TPA

After the cooking treatments described in Section 2.1 above, samples were placed in ice-cold water for 30 min then left in a 4 °C chiller for 24 h. The Warner-Bratzler peak shear force (WBSF; Newton) was measured following modified methods [17,21] using a Warner-Bratzler V-shaped shear blade attached to a texture analyser TA XT-plus 100 C (Stable Micro Systems, Surrey, UK). Five to six sub-samples (depending on sample size) of each sample with approximate dimensions of 1.0 × 1.0 × 5.0 cm (H × W × L) were analysed. TPA of cooked samples was measured by the method of De Huidobro et al. [45] on six to seven sub-samples from each sample using a 5-mm cylinder probe attached to a texture analyser (TA.C100 Plus Stable Micro Systems, UK). Approximately 1 cm^3^ sample was compressed to 75% of the height, the probe returned to initial height and a second compression cycle started after two seconds. Hardness, cohesiveness, adhesiveness, springiness, gumminess, and chewiness were calculated as described by Roldán et al. [46]. The details on the justification and parameters used for WBSF and TPA and the history of the development and use of WBSF has been provided by Warner, et al. [47].

#### 2.3.3. Total Collagen

Total collagen of all control and injected raw meat samples 24 h post injection was measured using the method of the Association of Official Agricultural Chemists [48], described in detail by Naqvi et al. [34], with minor modification. Briefly, the dried meat powder (100 mg) was hydrolysed in 3.5 M sulphuric acid (Sigma Aldrich, Castle Hill NSW, Australia) in a glass tube placed in an oven at 105 °C for 16 h. The approximate concentration of hydroxyproline was determined in dried meat powder using hydroxyproline standard solution and water (blank) to measure absorbance at 558 nm in a spectrophotometer (FLUO star Omega-415-0056, BMG LABTECH, Aylesbury, UK). The total collagen content of dried meat powder was calculated and expressed as mg/g of dried meat.

#### 2.3.4. Soluble Collagen

Heat soluble collagen content was measured for six replicates of each treatment in cooked samples, using the method of Christensen et al. [36], with minor modification, described in Naqvi et al. [34]. The method used the cooking loss fluid obtained from both control and injected samples after 8 h and 12 h of cooking. The hydroxyproline concentration was measured in cooking loss fluid using the colorimetric method and the amount of soluble collagen was calculated by multiplying the hydroxyproline concentration by a factor of 7.14. The heat soluble collagen determined in the cooking loss fluid was expressed as mg of soluble collagen/g cooked meat. 

### 2.4. Statistical Methods

All the physical, chemical and sensory data were analysed using linear mixed-effect models fitted using the restricted (or residual) maximum likelihood (REML) using nlme and emmeans packages in R (R version 3.5.2, R Core Team 2020, Vienna, Austria). The fixed effects for WBSF, TPA, TWC, soluble collagen, cooking loss, beef identity, metallic flavour, ginger flavour, juiciness, tenderness and texture consistency were injection treatment and cooking time whereas the random effects were carcass and location within the carcass and, for sensory evaluation, panel session, carcass number nested within the panel session, participant number, and participant number × panel session interaction. Total collagen measured in raw meat was analysed with the fixed effect specified as injection treatment, and the random effects as carcass and location within the carcass. The analysis of WBSF, soluble collagen, chewiness and adhesiveness, and the number of chews were performed on log-transformed data and the means estimated on log scale were back-transformed and presented in results. Texture description, a three-level categorical variable, was analysed using a set of three binomial mixed models using the glmer function of the lme4 package in R. The same fixed and random effects were specified as for the linear mixed models, and the emmeans package in the form of model-based probabilities was used for data output of mealy, mushy and other texture. The significance level was set at *P* = 0.05 to determine significant differences between means, and comparisons were made using a Fisher LSD approach using the cld function in the multcomp package in R. 

To investigate associations between the six sensory taste scores, correlations were obtained and plotted using the corrplot package in R, and a principal component analysis conducted (without re-scaling) using the prcomp function in R. The first two principal component (PC1, PC2) scores were plotted to investigate any clustering by injection treatment and cooking time. PC1 scores were analysed by a linear mixed model of the same form as specified for the separate sensory taste scores.

Following this, correlations were explored between the set of eight physical measurements (WBSF, hardness, adhesiveness, springiness, cohesiveness, gumminess, chewiness and total water content (TWC)) and the sensory scores. As TWC evaluation was not replicated, the average values of each of the other physical measurements were used (averaged across subsamples), resulting in 24 sets of physical observations. The sensory taste scores were similarly averaged and, following merging, resulted in 20 sets of combined physical-sensory variables. This dataset was initially explored through extended correlation analysis and plot, followed by canonical correlation analysis between the two sets, using the cc function in the Canonical Correlation Analysis (CCA) package and the cancor function in the candisc package in R. Variables were initially standardized to allow evaluation of the contribution of each physical and sensory variable to their respective canonical variables. Canonical correlations were tested and physical vs. sensory scores plotted to evaluate the associations.

## 3. Results

### 3.1. Sensory Evaluation

**Figure 1:**
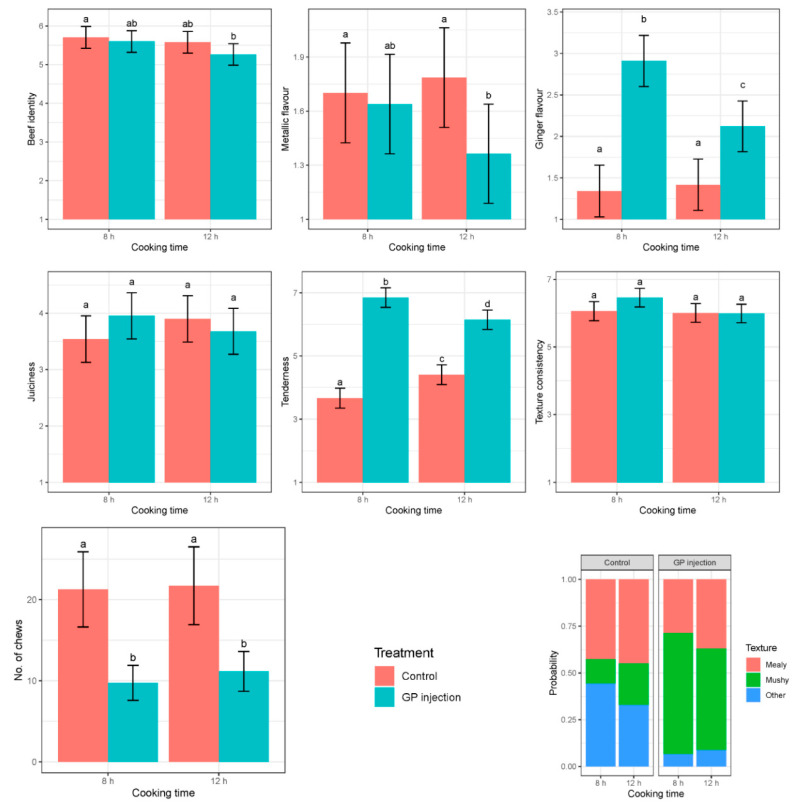
Effect of injection treatment (GP injection = Ginger powder solution containing zingibain injection, Control = without injection) and cooking time (8 h, 12 h) on sensory attributes of *M. biceps femoris* cooked at 65 °C and assessed through trained panelists. Eight-point scales were used for beef identity, metallic flavour and ginger flavour (1 = none, to 8 = extremely intense), juiciness (1 = extremely dry, to 8 = extremely juicy), tenderness (1 = extremely tough, to 8 = extremely tender), and texture consistency (1 = extremely inconsistent, to 8 = extremely uniform). Number of chews were counted by each panellist. Mean scores ± SE are presented in the bar chart for each sensory attribute and number of chews. Superscripts refer to significant differences between means. The texture description in the bottom right graph are based on a three-level categorical variable and are presented as a mean probability for the two injection treatments. A similar letter on each bar chat referring each attribute describes non-significance between treatments.

The samples had a moderate to slightly intense beef flavour intensity with mean scores of 5–6, assessed in all cooked samples at 65 °C for 8 h and 12 h of cooking (Figure 1). Beef flavour was not affected by injection treatment or cooking time (*p* > 0.1). 

Results showed a trend toward an interaction between injection treatment and cooking time (*p* = 0.084) on metallic flavour development in cooked meat. The trained sensory scores ranged from 1–2 (none to very bland) in all samples. GP-injected samples were assessed as having lower metallic flavour compared to no injection after12 h of cooking. 

Ginger flavour was found in all GP-injected samples within the mean score range of 2–3 (very bland to moderately bland). Ginger flavour was affected by an interaction between injection treatment and cooking time (*p* = 0.004) as shown in Figure 1. The ginger intensity was higher in GP-injected compared with control samples at both cooking times, but ginger flavour intensity was reduced after 12 h of cooking.

Juiciness was significantly impacted by the injection treatment × cooking time interaction (*p* = 0.032). GP-injected samples received higher juiciness scores at 8 h, whereas control samples had higher scores at 12 h cooking. 

Tenderness was greatly affected by both injection treatment and cooking time and their interaction (*p* < 0.001). GP-injected samples were substantially more tender compared with the controls, but tenderness increased with longer cooking time in control samples. 

Textural consistency was not affected by cooking time nor by injection treatment. However, the treatment had a significant effect (*p* < 0.001) on chewiness/number of chews required for the samples, with the GP-treated samples requiring fewer chews than the control samples at both cooking times. 

#### Correlations and Principal Component Analysis of Sensory Traits

**Figure 2:**
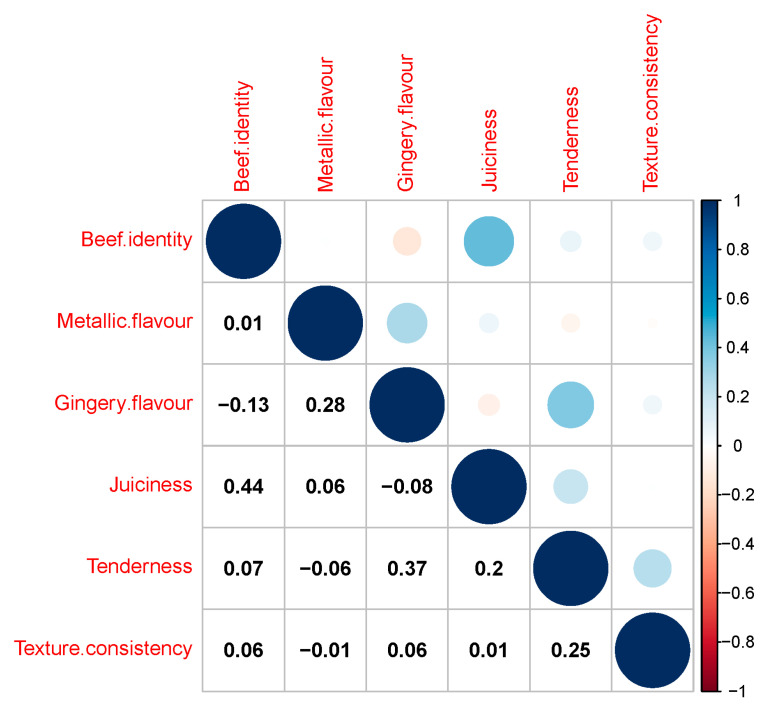
Plot of Pearson correlation coefficients of sensory attributes evaluated through trained panelists. Correlation values are shown below the diagonal and colour coding for size, and sign of correlations are shown above the diagonal. Correlations were calculated from sensory attributes of *M. biceps femoris* in control and ginger powder injection treated samples cooked for 8 h and 12 h. Blue dots indicate positive correlations and red dots represent negative correlations between the traits.

There were moderate positive correlations between juiciness and beef flavour identity (*r* = 0.44) and between tenderness and ginger flavour (*r* = 0.37), and low correlations between metallic flavour and ginger flavour (*r* = 0.28), between tenderness and texture consistency (*r* = 0.25) and between juiciness and tenderness (*r* = 0.20), as shown in Figure 2.

**Figure 3:**
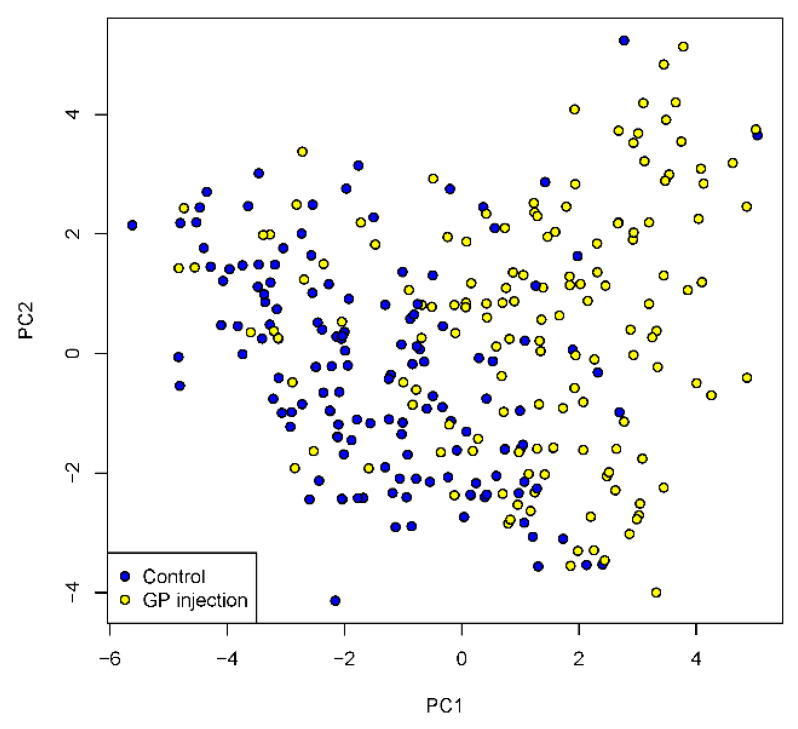
Scatter plot of principal component analysis (PCA), PC1 vs PC2 scores for sensory traits (beef flavour/identity, metallic flavour, ginger flavour, tenderness, juiciness, and texture consistency) of *M. biceps femoris* after injection treatment (GP injection = ginger powder injection containing zingibain and control = without injection), cooked at 65 °C for 8 h and 12 h under sous vide conditions. PCA separation was on injection treatment: yellow dots = injected samples; blue dots = control samples displayed in the plot.

A principal component analysis (PCA) was performed on the six sensory traits. The first PC (PC1) explained 34% of the variation, PC2 23%, PC3 16%, with the remaining four PCs explaining 27%. The loadings for PC1 and PC2 are listed in Table 3. Based on the loadings, PC1 can be interpreted as an overall measure of tenderness, texture consistency and juiciness together with ginger flavour, whereas, PC2 is a contrast between ginger flavour vs. beef flavour and juiciness. Figure 3 shows a scatter plot of PC1 vs. PC2 highlighting the separation between control and GP-injected samples. The GP-injected samples have higher PC1 values indicating increased tenderness, juiciness, and texture consistency along with ginger flavour. PC2 shows little separation between injection treatment and control samples. 

### 3.2. Physicochemical Measurements

#### 3.2.1. Instrumental Texture Profile

Peak shear force measured by WBSF was affected by an interaction between injection treatment and cooking time (*p* = 0.006). WBSF was significantly lower in GP-injected samples compared to controls, as shown in Table 4. Model-based means obtained for different textural parameters included in the TPA analysis are presented in Table 4.

#### 3.2.2. Total Water Content

**Figure 4:**
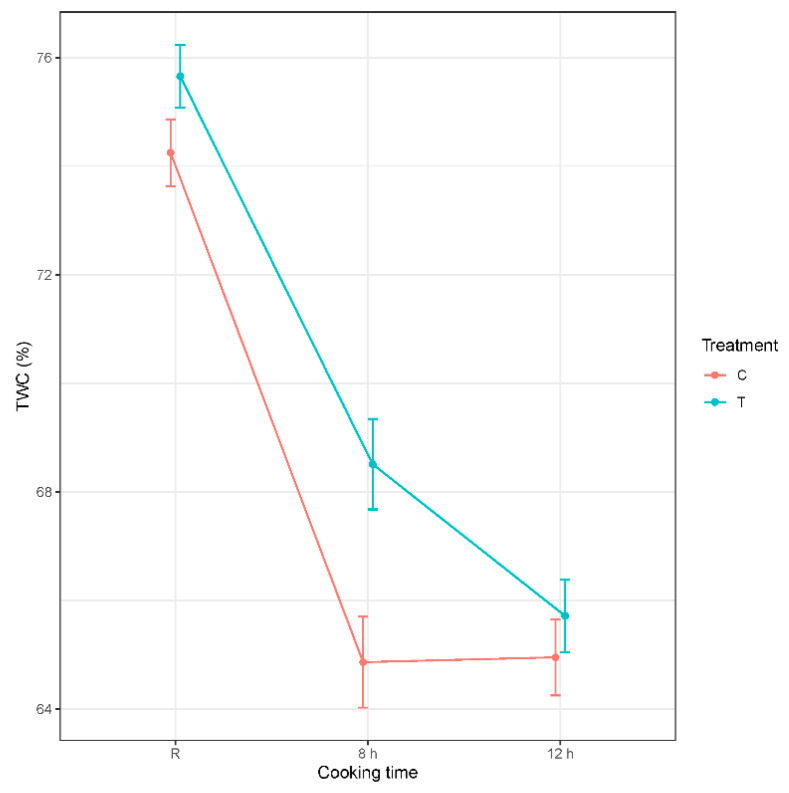
Effect of injection treatment (T = Injection with 2 g/L GP solution containing zingibain; C = control/without injection) and cooking time (8 h, 12 h) on total water content (TWC) of *M. biceps femoris* cooked at 65 °C under sous vide. Values are presented as mean ± SE for raw (R) and cooked meat samples.

The TWC (%) of raw BF was 74.9 ± 0.44 (mean ± SE) which was increased by injection treatment (*p* = 0.012), whereas the cooking of BF significantly (*p* < 0.001) reduced the total water content of the samples for both 8 h and 12 h cooking times from both treatments. There was no interaction found between injection treatments and cooking, as shown in Figure 4.

#### 3.2.3. Cooking Loss

An interaction between injection treatment and cooking time was observed (*p* = 0.05; Table 5) for cooking loss. Cooking loss was affected by cooking time with higher cooking loss after 12 h relative to 8 h of cooking. Cooking loss was higher in treated samples compared to controls at both cooking temperatures.

#### 3.2.4. Total Collagen

The total collagen of the raw BF was not affected by injection treatment (*p* = 0.26). The amount of collagen in injection treated samples, determined 24 h after injection treatment was 23 ± 4.1 and in control samples was 30 ± 4.1 (mg/g of dried meat) (mean ± SE). 

#### 3.2.5. Soluble Collagen

Soluble collagen was significantly higher in GP-injected cooked samples compared to control samples (*p* < 0.0001). There was a significant (*p* = 0.001) effect of cooking time on the amount of heat soluble collagen with higher concentrations in 12 h cooked samples compared to 8 h. There was no interaction between injection treatment and cooking time (*p* = 0.15), as shown in Table 6. However, in control samples, the soluble collagen increased with cooking time while there was no effect of cooking time in ginger-treated samples, with the same amount of heat soluble collagen found in both 8 h and 12 h cooked samples. 

#### 3.2.6. Canonical Correlation Analysis between Sensory Traits and Physical Measurements 

**Figure 5:**
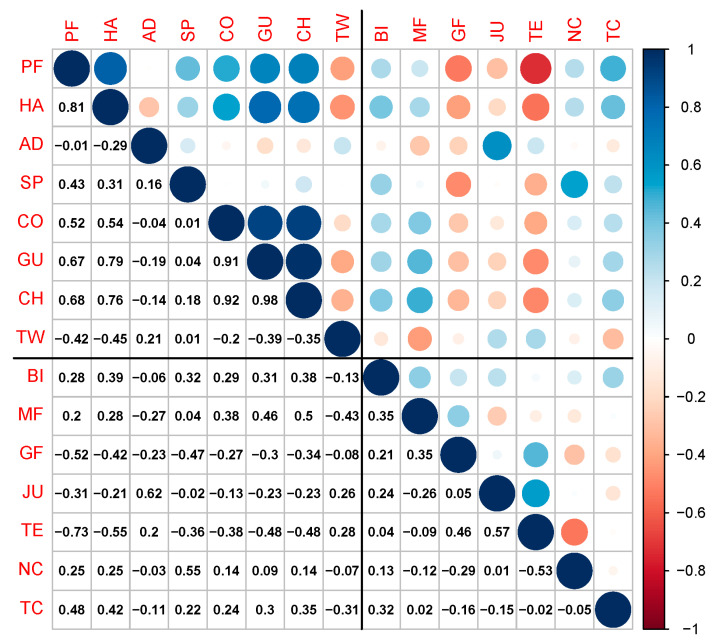
Plot of Pearson correlation coefficients (*r*) of sensory attributes (BI = beef identity, MF = metallic flavour, GF = ginger flavour, JU = juiciness, TE = tenderness, NC = number of chew, TC = textural consistency; evaluated through trained panellists) and physical measurements (PF = peak force, HA = hardness, AD = adhesiveness, SP = springiness, CO = cohesiveness, GU = gumminess, CH = chewiness and TW = total water content) for *M. biceps femoris* in control (without injection) and ginger powder injection samples cooked for 8 h and 12 h at 65 °C under sous vide. Correlation values are shown below the diagonal, and colour coding for size and sign of correlations are shown above the diagonal. Blue dots indicate positive correlations and red dot represents the negative correlations among the traits.

**Figure 6:**
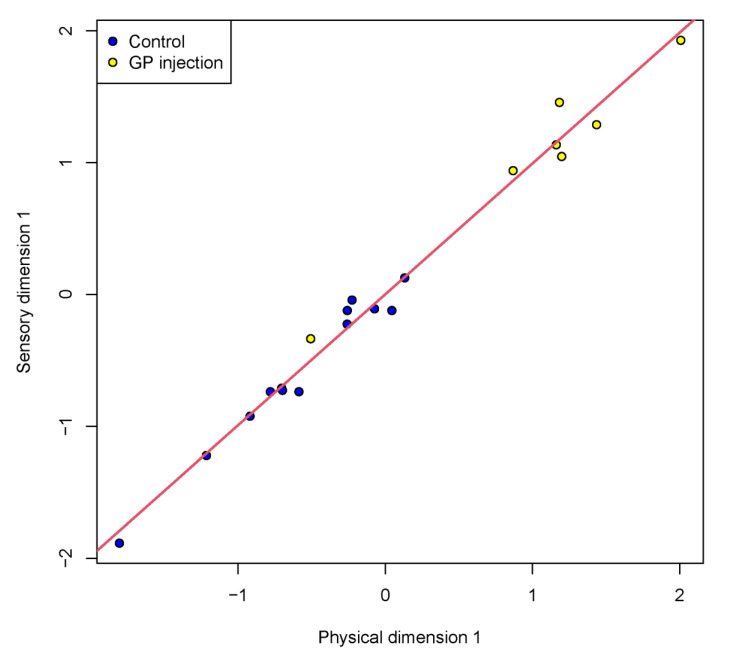
Canonical correlation plot showing first dimensions of physical (Warner-Bratzler shear force, hardness, adhesiveness, springiness, cohesiveness, gumminess, chewiness and total water content) and sensory traits (beef flavour/identity, metallic flavour, ginger flavour, tenderness, juiciness) of *M. biceps femoris* with injection treatment (control = without injection, GP injection = ginger powder injection containing zingibain) cooked at 65 °C for 8 h and 12 h under sous vide conditions. Control and GP-injected samples are displayed in the plot. Blue dots = Control, yellow dots = GP-injected samples.

Figure 5 shows the correlations between the eight physical measurements and the seven sensory traits. There are relatively high positive correlations among the physical measurement traits, although total water content is negatively correlated with most of the other physical measurement traits (*r* = −0.70). In contrast, there are relatively low correlations amongst the sensory traits, as noted previously. The cross-correlations between these two sets of traits are mostly moderate to high. Sensory tenderness had negative correlations with most of the physical measurement traits, particularly peak force (WBSF, *r* = −0.73), but there was a positive correlation with total water content (*r* = 0.57). Table 7 shows the loadings for the first pair of canonical variables for the canonical correlation analysis for the physical (*x*) and sensory (*y*) variables. From the loadings, the physical canonical variable is explained as a contrast between gumminess vs. chewiness, whereas the sensory canonical variate is explained as a contrast between tenderness and ginger flavour vs. beef flavour, metallic flavour and texture consistency. Figure 6 shows a plot of the sensory vs. physical canonical variables and displays a high canonical correlation (*r* = 0.99, *p* = 0.023). GP-injected samples had consistently higher scores than control samples for both physical and sensory measurements, which can be explained by the loadings as well as the individual sensory and physical variable findings reported above. 

## 4. Discussion

Our study demonstrated that GP-injected samples were more tender compared to control samples, with higher sensory tenderness scores of 6.85 ± 0.31 and 6.15 ± 0.30 for injected samples, compared to the control samples with scores of 3.66 ± 0.32 and 4.41 ± 0.32 (mean score ± SE) for 8 h and 12 h cooking times, respectively. These results are in agreement with the findings of Naveena and Mendiratta [29] in 4–5 year old buffalo meat, with a significant increase in sensory tenderness mean (± SE) scores in ginger extract treated samples (7.11 ± 0.09) compared to control samples (5.73 ± 0.11). The results of physical measurement of tenderness, WBSF and TPA provided additional objective evidence in support of the sensory results. Our results suggested that WBSF, hardness, cohesiveness, chewiness and gumminess were significantly reduced in GP-injected samples compared to control samples (Table 4), which is consistent with our previous study [21]. Our previous study [21] demonstrated that injecting water only has no effect on WBSF compared to control (no injection), demonstrating that our increased tenderness with GP injection is due to proteolysis, not muscle damage due to needle piercing. The use of ginger protease zingibain has been reported to improve tenderness in tough meat assessed by physical measurement and sensory evaluation. In the current study, control samples cooked for 8 h and 12 h were assessed as other/mealy in texture, whereas GP-injected samples were described as mushy, which is a reflection of over tenderization. A previous study has reported the mealy texture of the meat from older animals due to reduced juiciness of the meat [49]. A mushy and over-tenderised texture, which would be unacceptable to consumers, is also indicated by the very low WBSF for the GP-injected 12 h cooked samples. Hence, in contrast to our previous study [21], we achieved tender meat with lower cooking time of 8 h, with the use of GP-injection. However, the soft texture or over-tenderisation may be suitable for some specialised markets, for instance, the elderly population [50]. There are papers showing that people vary in their liking for tenderness and texture of the meat [47]. Elderly people desire a softer texture and often prefer very tender meat due to trouble chewing and swallowing difficulties [51,52].

Zingibain causes the breakdown of myofibrillar proteins and solubilization of collagen content, which enhances tenderization [53]. It acts more specifically on collagen due to substrate specificity for the amino acid proline on the collagen fibre, thus increasing soluble collagen contents of the cooked meat, which is positively associated with tenderness [54]. Our results revealed a significant amount of soluble collagen in the cooking fluid from GP-injected samples compared to control samples cooked at 65 °C for 8 h and 12 h. This is consistent with previous reports on tenderization of *M. pectoralis profundus* isolated from the beef brisket from Holstein steers with higher collagen solubility in ginger-treated samples compared to control [27]. Our results showed increased soluble collagen in the cooking fluid from both injection treated and control samples with longer heating. The heat-induced denaturation of collagen depends upon heating temperature and time. Purslow et al. [55] reported the denaturation of collagen protein and subsequent gelation between 60–70 °C. 

In the current study, all the sensory and physical measurements of tenderness were affected by longer cooking time. Tenderness of meat is associated with structural modification of myofibrillar proteins and connective tissue. Longer heating causes weakening of connective tissue, due to denaturation of proteins or solubilization of collagen [56]. A sensory study on low-temperature long time sous vide cooking by Mortensen et al. [37] reported improved tenderness of *semitendinosus* from bulls with increased cooking time (3–12 h) at 56 °C, 58 °C and 60 °C. Similarly, decreased shear force (WBSF) was reported by Christensen, Ertbjerg, Løje, Risbo, van den Berg and Christensen [36] in cow meat with longer cooking times ranging from 2.5–19 h using both 53 °C and 63 °C. Hence, these results are in agreement with the improved tenderness with trained sensory and decreased WBSF and TPA in the present study.

Juiciness is an important sensory attribute that is affected by the water holding capacity of the muscle, which affects the eating experience of the cooked meat. Juiciness and tenderness are closely associated and affected by cooking temperature and time [57,58]. In this study, the juiciness of the samples cooked at 65 °C for a longer time, regardless of the injection treatment, were assessed as less juicy through trained panellists, with mean scores between 3 and 4 (moderately dry to slightly dry). These results correspond to our TWC and cooking loss measurements. Previous studies showed that the TWC of the cooked meat was significantly affected by cooking temperature and time [34,56]. During heating, the fluid loss is related to myofibrillar shrinkage that initiates at 40 °C and intensifies with increasing temperature. The myofibrillar shrinkage reduces the ability of the muscle to hold water [59]. Increased temperature to 60–70 °C, causes contraction of peri-mysial connective tissue, which is associated with compression of muscle fibre bundles leading to the expulsion of fluids from muscle [55]. All these mechanisms explain the temperature effects on water loss and lower water contents of the cooked meat samples observed in the current study. 

In this study, reduced juiciness of samples with longer cooking time is consistent with previous studies on cooking duration in beef [60,61] and pork [62,63]. Although the TWC of raw injected samples was significantly higher than in the control samples, the cooking duration of 8 h and 12 h reduced the TWC of both control and injected samples, with a higher cooking loss in the injection-treated samples. This could most likely be explained by the higher water content of the injected samples but could also be explained by mechanical damage due to the process of repeated needling and excessive capillary action for ginger solution and water loss following injection of ginger solution. A value-added ready to use product for sous vide cooking, which might for example include mixed spices chosen according to the tastes of the consumers targeted for the product, could provide residual juice for the preparation of a tasty gravy to accompany the product upon serving. This would enhance the desirability of the product through flavour and, therefore, sensory acceptance. Our results showed that meat cooked at 65°C for 8 h and 12 h produced slightly intense to moderately intense beef flavour regardless of the treatments. Flavour of meat is impacted by the type and concentration of the volatile compounds produced during cooking [64]. The flavour in sous vide cooked beef mainly depends on cooking temperature [39]. Sous vide, a low-temperature cooking technology, produces less flavour in the meat [61]. Becker et al. [63] also found low flavour intensity in sous vide cooked pork if the cooking temperature was kept below 60 °C. Intense flavour is usually produced at above 70 °C in sous vide cooking [65]. This study found moderately intense flavour at a cooking temperature of 65 °C, in accordance with the above-mentioned studies for flavour intensity with temperature in sous vide cooking. 

Our results showed that very bland to moderately bland ginger flavour was present in all GP-injected samples assessed through the trained sensory panel representing diverse ethnicities and age groups. Previous studies have reported that the use of fresh ginger extract causes an off-flavour in the meat [9,31]. The ginger flavour is a mixture of spicy and sweet sensations and conveys a ‘warm’ impression with a strong pungent characteristic. Ginger contains phenolic compounds, carbohydrates, proteins, fibres and essential oils. The pungent flavour of ginger is produced by a group of phenolic alkanones such as gingerols and shagoals in the ginger rhizome [66]. Mainly, gingerols contribute to pungency in the crude ginger extract. The effect of ginger flavour can be minimized by using purification processes for protease extraction from the crude ginger rhizome. Previous studies found that the drying process reduces the gingerol content [67], while high-temperature processing converts gingerol to shagoals and zingerone, both of which are present in low concentration in crude ginger extract [68]. This study utilized the ginger powder containing partially purified proteases through drying aqueous extracts of ginger rhizomes, thus limiting the ginger flavour development in the meat. Our results suggest that using partially purified ginger powder solution may have an advantage over crude extract for the wider population, who do not always like a ginger flavour in meat. This is evident from the sensory evaluation scores (very bland to moderately bland) for ginger flavour (Figure 1).

Our results showed moderate to high correlations between sensory and physical traits which is in agreement with a previous study on tenderness in *M.* *longissimus thoracis* from bulls and steers, which reported moderate correlations between sensory and objective measurements [69]. In the current study, the TWC of the cooked meat and sensory juiciness are positively correlated (Figure 5). Sensory tenderness is negatively correlated with peak force (WBSF) and TPA parameters (hardness, cohesiveness, gumminess, springiness and chewiness). The tenderness and juiciness of cooked BF, evaluated through trained panellists, supported the physical measurements, indicating that these measurements are capable of predicting some aspects of the quality of the product. The flavour of the meat was only assessed by trained panellists and further analysis of the volatile profile contributing to beef flavour would be worthwhile.

## 5. Conclusions

The current study has demonstrated that the eating quality of tough meat from old animals can be enhanced by combining the use of sous vide cooking with ginger proteases. The physical and sensory characteristics of low-value beef, particularly tenderness, was improved with GP injection containing zingibain protease along with sous vide cooking, although GP injection and 12 h cooking resulted in mushy over-tenderised meat. Hence, a lower cooking time is required to reach acceptable tenderisation of very tough muscles when GP injection is used. The over-tenderised, mushy samples obtained with GP injection and sous vide cooking for 12 h are likely to be unacceptable to some consumers but may provide an opportunity for developing meat products for demographics such as the elderly who have difficulty chewing or swallowing. A low score for ginger flavour assessed by a trained sensory panel suggests that the use of purified ginger protease may be used without substantially masking the characteristic beef flavour. Our research clearly demonstrated a significant effect of protease enzyme and sous vide cooking on tenderness. The reduction in juiciness reported here could be increased by evaluating a mixture of spices having the potential to retain water: this requires further investigation. Future research is proposed on optimizing/adjusting the moisture contents with improved tenderness and higher sensory acceptability, which could be targeted for curry-based value-added products.

## Figures and Tables

**Table 1:** Selected beef attributes, description, and reference samples used to train the sensory panelists.

Attribute	Definition	References
Beef identity	Amount of beef flavour identity in the sample	Beef Broth = 5.0
		Beef brisket cooked to 71 °C = 8.0
Metallic flavour	Impression of slightly oxidized metal, such as iron, copper, and silver spoons	Strip steak cooked to 71 °C = 4.0
		Canned pineapple juice = 6.0
Ginger flavour	Slightly pungent impression/warm/spicy	Outside flat injected (10 g/L GP * solution) cooked at 65 °C = 8.0
		Outside flat injected (0.5 g/L GP solution cooked at 65 °C = 2.0
Tender	Easy to cut and chew	Tenderloin steak cooked to 65 °C = 8.0
		Strip steak cooked to 71 °C = 5.5
Chewy/tough	Requiring much chewing/tough	Eye of round steak cooked to 85 °C = 3.0
Inconsistent/patchy texture	Irregular or uneven in texture	Outside flat with uneven volume of GP injections per injection site (between 5–10 mL) cooked under sous vide at 65 °C = 8.0
Overall juiciness	Amount of liquid released from sample over the entire chewing	Strip steak cooked to 60 °C = 7.5
		Strip steak cooked to 71 °C = 5.0
		Strip steak cooked to 85 °C = 2–3

* GP = ginger powder.

**Table 2:** Scale description for scoring sensory attributes evaluated by trained sensory panelists.

Scale Point	Flavour Intensity	Juiciness	Tenderness	Texture Consistency
8	Extremely Intense	Extremely Juicy	Extremely Tender	Extremely Uniform
7	Very Intense	Very Juicy	Very Tender	Very Uniform
6	Moderately Intense	Moderately Juicy	Moderately Tender	Moderately Uniform
5	Slightly Intense	Slightly Juicy	Slightly Tender	Slightly Uniform
4	Slightly Bland	Slightly Dry	Slightly Tough	Slightly Inconsistent
3	Moderately Bland	Moderately Dry	Moderately Tough	Moderately Inconsistent
2	Very Bland	Very Dry	Very Tough	Very Inconsistent
1	None	Extremely Dry	Extremely Tough	Extremely Inconsistent

**Table 3:** Loadings for the first two principal components for the sensory traits.

Sensory Traits	PC1	PC2
Beef identity/flavour	0.092	−0.496
Metallic flavour	0.037	0.066
Ginger flavour	0.423	0.463
Juiciness	0.239	−0.731
Tenderness	0.824	0.023
Texture consistency	0.273	0.012

**Table 4:** Effect of injection treatment (IT; control = without injection; GP injection = ginger powder injection containing zingibain) and cooking time (T; 8 h, 12 h) on Warner-Bratzler shear force (WBSF), and texture profile parameters including hardness, cohesiveness, adhesiveness, springiness, chewiness, and gumminess of *M. biceps femoris* cooked at 65 °C under sous vide. Values are presented as mean ± SE. Superscripts refer to the significant differences of means (*p* < 0.05) within each row. *p* values for main effects (time and injection treatment) and their interaction are presented in the table.

Variables	Control		GP-Injection		*p* Values		
	8 h	12 h	8 h	12 h	Time (T)	Injection Treatment (IT)	T × IT
WBSF (N)	47.50 ± 4.11 ^a^	43.61 ± 3.56 ^a^	26.51 ± 3.18 ^b^	12.22 ± 1.17 ^c^	0.329	0.003	0.006
Hardness (N)	29.74 ± 1.54 ^a^	25.87 ± 1.53 ^a^	12.98 ± 2.50 ^b^	6.97 ± 1.77 ^b^	0.135	0.001	0.593
Cohesiveness (N/mm^2^)	0.42 ± 0.05 ^a^	0.25 ± 0.05 ^b^	0.21 ± 0.07 ^b^	0.16 ± 0.05 ^b^	0.042	0.045	0.342
Adhesiveness (N·s)	−0.02 ± 0.01 ^a^	−0.01 ± 0.01 ^a^	−0.02 ± 0.01 ^a^	−0.01 ± 0.01 ^a^	0.439	0.959	0.652
Springiness (cm)	0.49 ± 0.01 ^a^	0.45 ± 0.01 ^a^	0.50 ± 0.03 ^a^	0.36 ± 0.02 ^b^	0.123	0.769	0.057
Chewiness (N·s)	5.63 ± 2.323 ^a^	1.82 ± 0.75 ^ab^	0.68 ± 0.46 ^bc^	0.19 ± 0.09 ^c^	0.097	0.033	0.893
Gumminess (N/cm^2^)	12.62 ± 1.16 ^a^	6.92 ± 1.16 ^b^	2.12 ± 1.89 ^bc^	1.44 ± 1.34 ^c^	0.026	0.006	0.243

Hardness and chewiness of the cooked samples were significantly impacted only by injection treatment (both *p* < 0.01), whereas cohesiveness and gumminess were significantly affected by both injection treatment (*p* < 0.05 for both) and cooking time (*p* < 0.05 for both). However, there was no interaction between injection treatment and cooking time for these variables. There was an interaction between cooking time and injection treatment for springiness (*p* = 0.05), but adhesiveness was not affected by either cooking temperature or injection treatment, and nor was there any interaction. A similar letter on each row describes non-significance between treatments.

**Table 5:** Effect of injection treatment (GP injection = injection with ginger powder solution containing zingibain; Control = without injection) and cooking time (8 h, 12 h), on the cooking loss of *M. biceps femoris* cooked at 65 °C under sous vide. Data are presented as mean ± SE. Superscripts refer to significant differences in the mean values and a similar letter describes non-significance between treatments.

	Injection Treatment	Cooking Time	
		8 h	12 h
Cooking loss %	Control	32.5 ^a^ ± 0.83	34.5 ^b^ ± 0.70
	GP-injected	39.2 ^c^ ± 0.83	41.7 ^c^ ± 0.66

**Table 6:** Effect of injection treatment (GP injection = ginger powder injection containing zingibain; control = without injection) and cooking time (8 h, 12 h) on soluble collagen of *M. biceps femoris* cooked at 65 °C under sous vide. Values are presented as mean ± SE. Superscripts refer to significant differences in the mean values (*p* < 0.05). A similar letter describes non-significance between treatments.

	Injection Treatment	Cooking Time	
		8 h	12 h
Soluble collagen (mg soluble collagen/g raw meat)	Control	3.72 ± 0.43 ^a^	5.00 ± 0.57 ^b^
	GP-injection	14.05 ± 2.75 ^c^	17.72 ± 3.17 ^c^

**Table 7:** Canonical correlation loading of dimension 1 for both X and Y-axis. The X-axis represents the physical variables and Y-axis represents sensory variables of *M. biceps femoris* with injection treatment (control = without injection, GP injection = ginger powder injection containing zingibain) cooked at 65 °C for 8 h and 12 h under sous vide conditions. WBSF = Warner-Bratzler shear force, TWC = total water content.

Physical Variables	Canonical *x*-Loadings	Sensory Variables	Canonical *y*-Loadings
WBSF	−0.537	Beef flavour	−0.321
Hardness	−0.411	Metallic flavour	−0.392
Adhesiveness	0.151	Ginger flavour	0.364
Springiness	0.245	Juiciness	0.014
Cohesiveness	−0.009	Tenderness	0.440
Gumminess	2.972	No of chews	0.045
Chewiness	−3.026	Texture consistency	−0.421
TWC	0.074		

## Data Availability

The datasets generated for this study are available on request to the corresponding author.

## References

[1] Polkinghorne R., Thompson J. (2010). Meat standards and grading: A world view. Meat Sci..

[2] Grunert K.G., Bredahl L., Brunsø K. (2004). Consumer perception of meat quality and implications for product development in the meat sector—A review. Meat Sci..

[3] Hocquette J.-F., Botreau R., Picard B., Jacquet A., Pethick D.W., Scollan N.D. (2012). Opportunities for predicting and manipulating beef quality. Meat Sci..

[4] Savell J., Lorenzen C., Neely T., Miller R., Tatum J., Wise J., Taylor J., Buyck M., Reagan J. (1999). Beef customer satisfaction: Cooking method and degree of doneness effects on the top sirloin steak. J. Anim. Sci..

[5] Shackelford S.D. (2001). Consumer impressions of tender select beef. J. Anim. Sci..

[6] Lyford C.P., Thompson J.M., Polkinghorne R., Miller M.F., Nishimura T., Neath K., Allen P., Belasco E.J. (2010). Is willingness to pay (WTP) for beef quality grades affected by consumer demographics and meat consumption preferences?. Australas. Agribus. Rev..

[7] Boleman S., Boleman S.L., Miller R., Taylor J., Cross H., Wheeler T., Koohmaraie M., Shackelford S., Miller M., West R. (1997). Consumer evaluation of beef of known categories of tenderness. J. Anim. Sci..

[8] Therkildsen M., Stolzenbach S., Byrne D.V. (2011). Sensory profiling of textural properties of meat from dairy cows exposed to a compensatory finishing strategy. Meat Sci..

[9] Sullivan G.A., Calkins C.R. (2010). Application of exogenous enzymes to beef muscle of high and low-connective tissue. Meat Sci..

[10] Purslow P. (2014). New developments on the role of intramuscular connective tissue in meat toughness. Annu. Rev. Food Sci. Technol..

[11] Harris P.V. (1976). Structural and other aspects of meat tenderness. J. Texture Stud..

[12] Warner R.D., Greenwood P.L., Pethick D.W., Ferguson D.M. (2010). Genetic and environmental effects on meat quality. Meat Sci..

[13] Purslow P.P. (2005). Intramuscular connective tissue and its role in meat quality. Meat Sci..

[14] Ashie I.N.A., Sorensen T.L., Nielsen P.M. (2002). Effects of papain and a microbial enzyme on meat proteins and beef tenderness. J. Food Sci..

[15] Arshad M.S., Kwon J.-H., Imran M., Sohaib M., Aslam A., Nawaz I., Amjad Z., Khan U., Javed M. (2016). Plant and bacterial proteases: A key towards improving meat tenderization, a mini review. Cogent Food Agric..

[16] Christensen M., Tørngren M., Gunvig A., Rozlosnik N., Lametsch R., Karlsson A., Ertbjerg P. (2009). Injection of marinade with actinidin increases tenderness of porcine M. biceps femorisand affects myofibrils and connective tissue. J. Sci. Food Agric..

[17] Zhu X., Kaur L., Staincliffe M., Boland M. (2018). Actinidin pretreatment and sous vide cooking of beef brisket: Effects on meat microstructure, texture and in vitro protein digestibility. Meat Sci..

[18] Ha M., Bekhit A., Carne A., Hopkins D. (2013). Comparison of the proteolytic activities of new commercially available bacterial and fungal proteases toward meat proteins. J. Food Sci..

[19] Bekhit A.A., Hopkins D.L., Geesink G., Bekhit A.A., Franks P. (2014). Exogenous proteases for meat tenderization. Crit. Rev. Food Sci. Nutr..

[20] Gagaoua M., Dib A.L., Lakhdara N., Lamri M., Botineştean C., Lorenzo J.M. (2021). Artificial meat tenderization using plant cysteine proteases. Curr. Opin. Food Sci..

[21] Naqvi Z.B., Campbell M.A., Latif S., Thomson P.C., McGill D.M., Warner R.D., Friend M.A. (2021). Improving tenderness and quality of M. *biceps femoris* from older cows through concentrate feeding, zingibain protease and sous vide cooking. Meat Sci..

[22] Thompson E.H., Wolf I.D., Allen C.E. (1973). Ginger rhizome: A new source of proteolytic enzyme. J. Food Sci..

[23] Saranya S., Santhi D., Kalaikannan A. (2016). Ginger as a tenderizing agent for tough meats—A review. J. Livest. Sci..

[24] Cruz P.L., Panno P.H.C., Giannotti J.D.G., Carvalho R.V.d., Roberto C.D. (2020). Effect of proteases from ginger rhizome on the fragmentation of myofibrils and tenderness of chicken breast. LWT Food Sci. Technol..

[25] Lee Y.B., Sehnert D.J., Ashmore C.R. (1986). Tenderization of meat with ginger rhizome protease. J. Food Sci..

[26] He F.-Y., Kim H.-W., Hwang K.-E., Song D.-H., Kim Y.-J., Ham Y.-K., Kim S.-Y., Yeo I.-J., Jung T.-J., Kim C.-J. (2015). Effect of ginger extract and citric acid on the tenderness of duck breast muscles. Korean J. Food Sci. Anim. Resour..

[27] Moon S.S. (2018). Effect of proteolytic enzymes and ginger extract on tenderization of m. *Pectoralis profundus* from holstein steer. Korean J. Food Sci. Anim. Resour..

[28] Naveena B.M., Mendiratta S.K., Anjaneyulu A.S.R. (2004). Tenderization of buffalo meat using plant proteases from *Cucumis trigonus Roxb* (Kachri) and *Zingiber* officinale roscoe (Ginger rhizome). Meat Sci..

[29] Naveena B.M., Mendiratta S.K. (2004). The tenderization of buffalo meat using ginger extract. J. Muscle Foods.

[30] Pawar V.D., Mule B.D., Machewad G.M. (2007). Effect of marination with ginger rhizome extract on properties of raw and cooked chevon. J. Muscle Foods.

[31] Bhaskar N., Sachindra N.M., Modi V.K., Sakhare P.Z., Mahendrakar N.S. (2006). Preparation of proteolytic activity rich ginger powder and evaluation of its tenderizing effect on spent-hen muscles. J. Muscle Foods.

[32] Schellekens M. (1996). New research issues in sous-vide cooking. Trends Food Sci. Technol..

[33] Baldwin D. (2012). Sous vide cooking: A review. Int. J. Gastron. Food Sci..

[34] Naqvi Z.B., Thomson P.C., Ha M., Campbell M.A., McGill D.M., Friend M.A., Warner R.D. (2021). Effect of sous vide cooking and ageing on tenderness and water-holding capacity of low-value beef muscles from young and older animals. Meat Sci..

[35] Bhat Z.F., Morton J.D., Zhang X., Mason S.L., Bekhit A.E.-D.A. (2020). Sous-vide cooking improves the quality and in-vitro digestibility of Semitendinosus from culled dairy cows. Food Res. Int..

[36] Christensen L., Ertbjerg P., Løje H., Risbo J., van den Berg F.W.J., Christensen M. (2013). Relationship between meat toughness and properties of connective tissue from cows and young bulls heat treated at low temperatures for prolonged times. Meat Sci..

[37] Mortensen L., Frøst M., Skibsted L., Risbo J. (2012). Effect of time and temperature on sensory properties in low-temperature long-timesous-vide cooking of beef. J. Culin. Sci. Technol..

[38] Ruiz J., Calvarro J., Sánchez del Pulgar J., Roldán M. (2013). Science and technology for new culinary techniques. J. Culin. Sci. Technol..

[39] Christensen L., Gunvig A., Torngren M.A., Aaslyng M.D., Knochel S., Christensen M. (2012). Sensory characteristics of meat cooked for prolonged times at low temperature. Meat Sci..

[40] Sun S., Rasmussen F.D., Cavender G.A., Sullivan G.A. (2019). Texture, color and sensory evaluation of sous-vide cooked beef steaks processed using high pressure processing as method of microbial control. LWT Food Sci. Technol..

[41] Botinestean C., Hossain M., Mullen A.M., Kerry J.P., Hamill R.M. (2021). The influence of the interaction of sous-vide cooking time and papain concentration on tenderness and technological characteristics of meat products. Meat Sci..

[42] Lucherk L., O’Quinn T., Legako J., Rathmann R., Brooks J., Miller M. (2016). Consumer and trained panel evaluation of beef strip steaks of varying marbling and enhancement levels cooked to three degrees of doneness. Meat Sci..

[43] AMSA (2015). Research Guidelines for Cookery, Sensory Evaluation, and Instrumental Tenderness Measurements of Meat.

[44] Oillic S., Lemoine E., Gros J.-B., Kondjoyan A. (2011). Kinetic analysis of cooking losses from beef and other animal muscles heated in a water bath—Effect of sample dimensions and prior freezing and ageing. Meat Sci..

[45] De Huidobro F.R., Miguel E., Blázquez B., Onega E. (2005). A comparison between two methods (Warner–Bratzler and texture profile analysis) for testing either raw meat or cooked meat. Meat Sci..

[46] Roldán M., Antequera T., Martín A., Mayoral A.I., Ruiz J. (2013). Effect of different temperature–time combinations on physicochemical, microbiological, textural and structural features of sous-vide cooked lamb loins. Meat Sci..

[47] Warner R., Miller R., Ha M., Wheeler T.L., Dunshea F., Li X., Vaskoska R., Purslow P., Wheeler T. (2021). Meat tenderness: Underlying mechanisms, instrumental measurement, and sensory assessment. Meat Muscle Biol..

[48] AOAC (2000). Official Methods of Analysis of the Association of Official Analytical Chemists.

[49] Shorthose W.R., Harris P.V. (1990). Effect of animal age on the tenderness of selected beef muscles. J. Food Sci..

[50] Mena B., Ashman H., Dunshea F.R., Hutchings S., Ha M., Warner R.D. (2020). Exploring meal and snacking behaviour of older adults in Australia and China. Foods.

[51] Felderhoff C., Lyford C., Malaga J., Polkinghorne R., Brooks C., Garmyn A., Miller M. (2020). Beef quality preferences: Factors driving consumer satisfaction. Foods.

[52] Botinestean C., Keenan D.F., Kerry J.P., Hamill R.M. (2016). The effect of thermal treatments including sous-vide, blast freezing and their combinations on beef tenderness of *M. semitendinosus* steaks targeted at elderly consumers. LWT Food Sci. Technol..

[53] Ha M., Bekhit A.E.-D.A., Carne A., Hopkins D.L. (2012). Characterisation of commercial papain, bromelain, actinidin and zingibain protease preparations and their activities toward meat proteins. Food Chem..

[54] Kim M., Hamilton S., Guddat L., Overall C. (2007). Plant collagenase: Unique collagenolytic activity of cysteine proteases from ginger. Biochim. Et Biophys. Acta.

[55] Purslow P.P., Oiseth S., Hughes J., Warner R.D. (2016). The structural basis of cooking loss in beef: Variations with temperature and ageing. Food Res. Int..

[56] Dominguez Hernandez E., Salaseviciene A., Ertbjerg P. (2018). Low-temperature long-time cooking of meat: Eating quality and underlying mechanisms. Meat Sci..

[57] Tornberg E. (2005). Effects of heat on meat proteins—Implications on structure and quality of meat products. Meat Sci..

[58] Huff-Lonergan E., Lonergan S.M. (2005). Mechanisms of water-holding capacity of meat: The role of postmortem biochemical and structural changes. Meat Sci..

[59] Hughes J.M., Oiseth S.K., Purslow P.P., Warner R.D. (2014). A structural approach to understanding the interactions between colour, water-holding capacity and tenderness. Meat Sci..

[60] Modzelewska-Kapituła M., Dąbrowska E., Jankowska B., Kwiatkowska A., Cierach M. (2012). The effect of muscle, cooking method and final internal temperature on quality parameters of beef roast. Meat Sci..

[61] Ayub H., Ahmad A. (2019). Physiochemical changes in sous-vide and conventionally cooked meat. Int. J. Gastron. Food Sci..

[62] Aaslyng M.D., Bejerholm C., Ertbjerg P., Bertram H.C., Andersen H.J. (2003). Cooking loss and juiciness of pork in relation to raw meat quality and cooking procedure. Food Qual. Prefer..

[63] Becker A., Boulaaba A., Pingen S., Krischek C., Klein G. (2016). Low temperature cooking of pork meat—Physicochemical and sensory aspects. Meat Sci..

[64] Hwang Y.-H., Ismail I., Joo S.-T. (2020). Identification of umami taste in sous-vide beef by chemical analyses, equivalent umami concentration, and electronic tongue system. Foods.

[65] Roldán M., Ruiz J., del Pulgar J.S., Pérez-Palacios T., Antequera T. (2015). Volatile compound profile of sous-vide cooked lamb loins at different temperature–time combinations. Meat Sci..

[66] Ravindran P. (2017). The Encyclopedia of Herbs and Spices.

[67] Bartley J.P., Jacobs A.L. (2000). Effects of drying on flavour compounds in Australian-grown ginger (Zingiber officinale). J. Sci. Food Agric..

[68] Menon A.N., Padmakumari K., Kutty B.S., Sumathikutty M., Sreekumar M., Arumugham C. (2007). Effects of processing on the flavor compounds of indian fresh ginger (*Zingiber Officinale* Rose.). J. Essent. Oil Res..

[69] Peachey B.M., Purchas R.W., Duizer L.M. (2002). Relationships between sensory and objective measures of meat tenderness of beef m. longissimus thoracis from bulls and steers. Meat Sci..

